# Cellular regeneration strategies for macular degeneration: past, present and future

**DOI:** 10.1038/s41433-018-0061-z

**Published:** 2018-03-05

**Authors:** Valeria Chichagova, Dean Hallam, Joseph Collin, Darin Zerti, Birthe Dorgau, Majed Felemban, Majlinda Lako, David H. Steel

**Affiliations:** 1 Institute of Genetic Medicine, Newcastle University, Newcastle upon Tyne, UK; 2 Sunderland Eye Infirmary, Queen Alexandra Road, Sunderland, UK

## Abstract

Despite considerable effort and significant therapeutic advances, age-related macular degeneration (AMD) remains the commonest cause of blindness in the developed world. Progressive late-stage AMD with outer retinal degeneration currently has no proven treatment. There has been significant interest in the possibility that cellular treatments may slow or reverse visual loss in AMD. A number of modes of action have been suggested, including cell replacement and rescue, as well as immune modulation to delay the neurodegenerative process. Their appeal in this enigmatic disease relate to their generic, non-pathway-specific effects. The outer retina in particular has been at the forefront of developments in cellular regenerative therapies being surgically accessible, easily observable, as well as having a relatively simple architecture. Both the retinal pigment epithelium (RPE) and photoreceptors have been considered for replacement therapies as both sheets and cell suspensions. Studies using autologous RPE, and to a lesser extent, foetal retina, have shown proof of principle. A wide variety of cell sources have been proposed with pluripotent stem cell-derived cells currently holding the centre stage. Recent early-phase trials using these cells for RPE replacement have met safety endpoints and hinted at possible efficacy. Animal studies have confirmed the promise that photoreceptor replacement, even in a completely degenerated outer retina may restore some vision. Many challenges, however, remain, not least of which include avoiding immune rejection, ensuring long-term cellular survival and maximising effect. This review provides an overview of progress made, ongoing studies and challenges ahead.

## Introduction

Age-related macular degeneration (AMD) is the commonest cause of blindness in the developed world. The number of patients with currently non-treatable AMD is staggering, being responsible for approximately half of the 370,000 people registered as blind or partially sighted in the UK alone [1]. Late-stage AMD affects over 2.4% of the adult population over 50 and 12% of those over 80 years. The number of AMD cases is predicted to rise by one-third over the next decade, totalling nearly 700,000 in the UK by 2020 and 1,300,000 by 2050, with healthcare costs rising to £16.4 billion during 2010–2020 [2]. Each year in the UK, it is estimated that ~70,000 patients present with late AMD; half with wet disease and half with dry [3]. AMD is a worldwide disease and globally it is thought to affect over 8 million people.

**Fig. 1:**
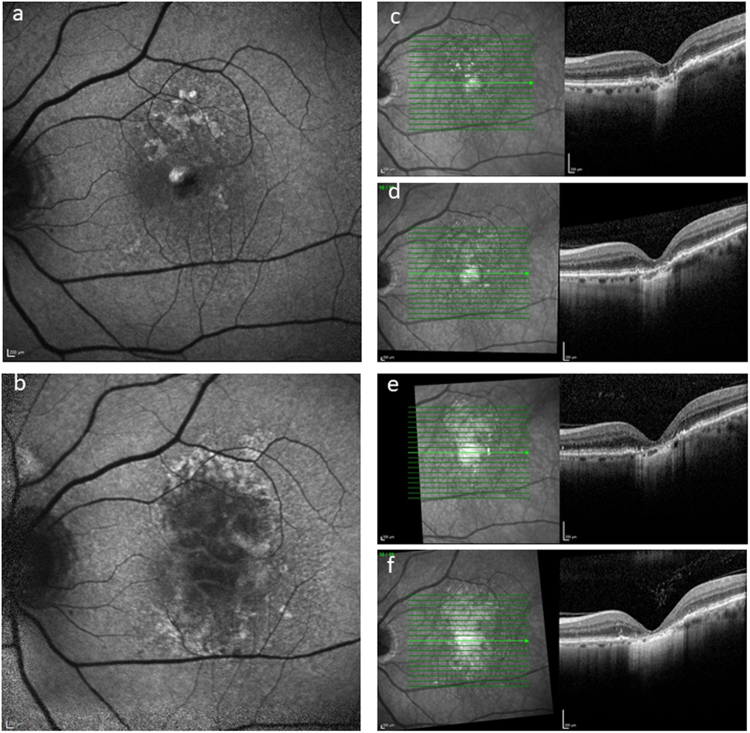
A 76-year-old female patient presenting with dry AMD. First seen in 2013 with a visual acuity of logMAR 0.3 and small areas of paracentral RPE atrophy with surrounding drusen (**a**). Her vision slowly deteriorated to logMAR 1.0 over 3 years with increasing central geographic atrophy (**b**). Progression of central outer retinal atrophy shown on spectral domain optical coherence tomography (SDOCT) (**c**–**f**)

AMD is manifested fundoscopically in the early and intermediate stages by the appearance of yellowish subretinal deposits, called drusen deep to the retinal pigment epithelium (RPE) in the macular retina. At this stage, the effect on vision is relatively mild, although acuity in low-contrast conditions is frequently affected. At least 15% of patients progress however to the more advanced ‘wet’ and ‘dry’ forms of the disease. Dry AMD is characterised by degeneration of the RPE and subsequently the overlying photoreceptors. ‘Wet’ AMD is characterised by aberrant choroidal blood vessel growth beneath or through the RPE, affecting the function of the overlying neurosensory retina by vascular leak, haemorrhage and fibrosis with subsequent outer retinal degeneration. Treatments are available and evolving for wet AMD, most notably, anti-vascular endothelial growth factor (VEGF) treatment [4]. However, there are, as yet, no effective treatments to prevent progression of the underlying disease processes and advancement of dry AMD (Fig. 1). This partly relates to the fact that the disease process is complex and multifaceted, with both environmental and genetic risk associations and the interplay of a variety of cellular abnormalities, including impaired autophagy and chronic innate immune activation [5]. Similarly, outer retinal degenerations caused by monogenetic defects are now the commonest causes of blindness in the working age group in the UK, with the macular dystrophy, Stargardt disease being one of the commonest [1]. It has several similarities to atrophic AMD, and although many approaches are being considered, none are licensed and proven as yet [6].

Gene therapy and a variety of other therapies are being investigated as possible treatments for these diseases, but they are unlikely to restore vision once photoreceptors loss has occurred nor do they aim to restore the RPE [7]. Electronic retinal interface devices show promise for navigative vision in patients with advanced disease, but the level of resolution achievable is likely a long way from foveal vision [8]. Similarly, although optogenetic approaches, with the induced expression of variety of light sensitive molecules most notably Channelrhodopsin on ganglion cells (NCT02556736, NCT03326336), are being investigated, high-resolution vision is again unlikely.

**Fig. 2:**
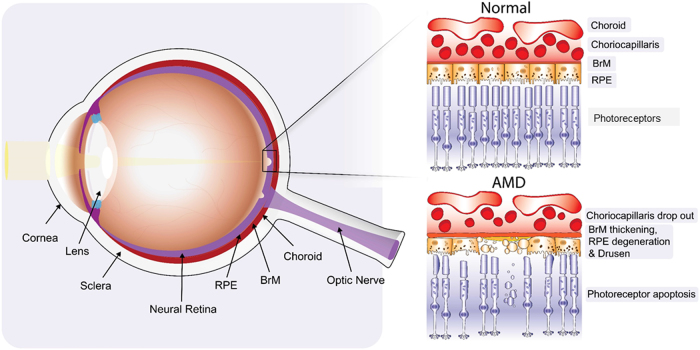
Schematic diagram illustrating normal retina contrasted with the changes observed in dry AMD

A variety of cellular regenerative therapies with a range of cell types are being studied for the treatment of AMD and other outer retinal conditions. Transplantation is perhaps the most obvious mode of action and the idea of ‘young’ cells restoring and replacing the old is a particularly alluring concept. In advanced AMD, despite extensive outer retinal degeneration, the inner retina with its intricate neural connectivity and output via the ganglion cells to the brain, is anatomically intact, allowing the realistic possibility that cell replacement in the outer retina may restore vision [9]. AMD is thought to be principally a disease of the RPE/Bruch’s membrane (BrM)/choriocapillaris (CC) complex initially and therefore in patients who present with wet AMD of recent onset where there are preserved photoreceptors, RPE (+/− BrM+/− CC) replacement alone may be enough. Conversely, in those with dry AMD and wet disease of longer duration, there will be varying degrees of accompanying photoreceptor atrophy (Fig. 2).

**Fig. 3:**
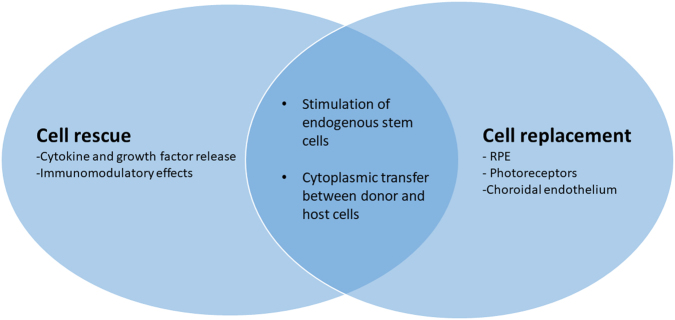
Potential multiple and overlapping modes of action of cellular therapies for AMD

However, the appeal of cellular therapies also rests in their potential multiple mechanisms of action and their generic, non-disease-specific effects that are particularly attractive in a disease like AMD. Cell therapies can rescue host cells by the production of cytokines and neurotrophic factors as well as altering the neurodegenerative process by immunomodulatory effects [10, 11]. Treatment with mesenchymal-derived cells, including mesenchymal stem cells (MSCs), and umbilical tissue cells are specifically being evaluated for these effects. Some of these cells may also debatably stimulate the endogenous activation of in situ stem cells, and hence their action may be multi-fold. Similarly, recent evidence has also shown that transplanted photoreceptor precursor cells can restore cellular and visual function by cytoplasmic transfer to host cells rather than purely cell integration and potentially the neuroprotective effects of cellular therapies may be among their most important function, including the stimulation of intrinsic retinal repair processes [12, 13] (Fig. 3).

Progress has been rapid, and transplanted cells from a range of types and sources have been shown to restore visual function in animal models of human disease, and some are in early-phase human trials. Indeed the eye is at the forefront of cell-based therapies, being an ideal site to evaluate their effects. This is particularly true for outer retinal diseases, where the confined, relatively immune privileged, easily observed and surgically accessible space between the photoreceptors and RPE is a particularly tractable place to inject and evaluate treatments. The anatomical connections required for proposed cellular transplantation are relatively simple for both RPE cells and photoreceptors relative to other central nervous system locations, and retinal imaging and functional assessment is at an advanced level with cellular level imaging possible. The number of cells required to provide measurable visual function is potentially low, with as few as 25,000 cells restoring some vision in murine disease, reducing the antigenic load and cell production logistics [14]. Intravitreal injections are now an everyday part of clinical care and cells delivered in this way could also have outer retinal effects by paracrine-mediated mechanisms easing logistic concerns.

The history and ongoing efforts to produce an effective method of regenerating the degenerate outer retina, and in particular AMD is a compelling story covered by several excellent reviews, which we draw upon [15–21]. Our aim in this further review is to outline the wide-ranging progress being made in the field and the future challenges that lie ahead.

## Historical perspective and background

### RPE transplantation

The RPE is the name given to the monolayer of hexagonal-shaped pigmented epithelial cells that underlie the neurosensory retina. They grow on a specialised basement membrane, BrM and tight junctions between the RPE cells form the outer retinal blood barrier. The RPE is vital to the survival and function of the photoreceptors, and in addition to phagocytosing photoreceptor outer segments and recycling retinol as part of the visual cycle, it has several additional key functions, including polarised growth factor secretion, nutrient transport, stray light absorption and antioxidant functions. With age, the ability of the RPE to phagocytose and remove toxic by-products of phototransduction reduces, resulting in the accumulation of toxic material that in turn reduces the ability of the cell to function optimally. This process, coupled with other age-associated changes in the RPE and BrM are key stages in the development of AMD [5]. Clinical observations have shown that in vivo the RPE has very limited ability to regenerate and consequently degeneration of these cells leads to photoreceptor death and irreversible blindness.

There are two potential roles of RPE cell transplantation in AMD, namely replacement of RPE function and trophic support to dying cells (reviewed by Alexander et al. [22]). RPE transplantation was first shown to successfully rescue photoreceptor death in rats with a defect in the ability of RPE cells to phagocytose photoreceptor outer segment discs secondary to a mutation in the transmembrane proto-oncogene tyrosine-protein kinase MER gene (Royal College of Surgeons (RCS) rats) in 1988. As well as phagocytosing shed outer segments RPE cells produce several neurotrophic factors [23] and photoreceptor rescue can occur distant to the transplanted cells, up to 1400 microns away in one study [24]. They also produce VEGF, which is trophic for the CC endothelium. Recent research has shown that loss of the endothelial cells of the CC is one of the earliest detectable events in AMD and because the RPE relies on the CC for metabolic support, this loss may be the trigger for progression to more advanced stages (reviewed by Chirco et al. [25]). RPE transplant may potentially thus trigger choroidal vascular endothelial regeneration.

**Fig. 4:**
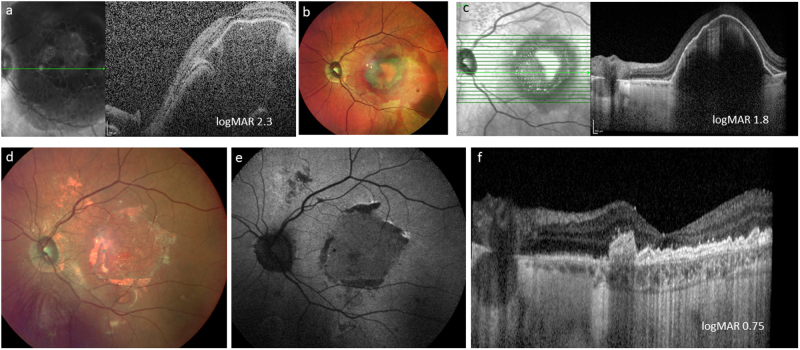
77-year-old female patient presenting with large submacular haemorrhage secondary to acute wet AMD (**a**). She initially underwent subretinal haemorrhage displacement surgery with vitrectomy, subretinal tissue plasminogen activator and ranibizuamb and air that although successful in terms of haemorrhage displacement revealed a large submacular scar (colour image (**b**), and SDOCT (**c**). The patient then underwent subretinal choroidal neovascular membrane removal, and peripheral large RPE/choroidal graft with a 200 degree temporal retinotomy. Postoperative appearance (**d**), with corresponding autofluorescent image showing uniform normal autofluorescence (**e**) and SDOCT with a perfused choroidal appearance visible (**f**)

**Fig. 5:**
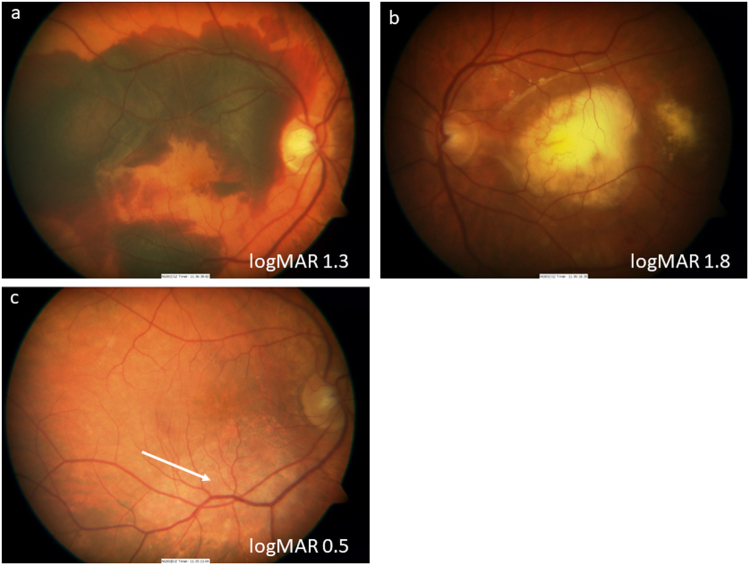
A 78-year-old male patient presenting with large submacular haemorrhage and extensive choroidal neovascular membrane in his right eye (**a**), having already lost vision in his left eye with an established disciform scar (**b**). Patient underwent macular relocation surgery with a 360 degree peripheral retinotomy and CNVM removal, and subsequent counter rotation surgery with visual improvement (**c**). Note scar (white arrow) from previous CNVM now eccentric to fovea

The concept of RPE transplantation was quickly transferred to human studies with autologous RPE sheet transplantation in advanced wet AMD. Both macular relocation surgery, whereby the neurosensory retina is relocated from a fresh non-diseased area of RPE, and autologous RPE patches from the peripheral retina have demonstrated that, in selected cases, it is possible to restore vision long term in patients with recent onset wet AMD, where irreversible photoreceptor degeneration has not yet occurred [26–32] (Figs. 4 and 5).

### Photoreceptor transplantation

When irreversible photoreceptor loss has occurred in AMD, combined RPE and photoreceptor transplantation will be required. As mentioned, despite profound outer retinal degeneration, the neurons of the inner nuclear layer survive for extended time periods after photoreceptor degeneration despite significant dendritic retraction and circuit remodelling. Studies with retinal prosthetic devices have shown that patients appear to retain a retinotopic map even after extensive outer retinal degeneration if the retina can be stimulated appropriately. Replacing degenerated photoreceptors would be one way to do this. Transplanted cells would only need to make one synaptic connection to the bipolar cells. The synaptic remodelling that developed during regeneration would need to reverse; however, it is known that plasticity exists in this regard [33].

There have been two approaches to photoreceptor replacement considered, namely disassociated photoreceptor cells delivered as a suspension and retinal sheet or micro-aggregate transplantation (reviewed by Seiler and Aramant [34]).

#### Transplantation of dissociated cells

The first transplants with disassociated neural retinal cells into the subretinal space were carried out in light damaged and RCS rats [35, 36]. Transplanted cells degenerated over time without benefit. A key discovery, however, was made in 2006, when young and immediately post-mitotic photoreceptor precursors rather than retinal progenitor cells (RPC), which are still in the proliferation phase or mature adult cells, were identified as the most successful cell-type for transplantation. Experiments showed that these cells were capable of long-term survival, maturation and visual improvement following transplantation into murine models of retinal degeneration [14, 37–40]. Adult photoreceptors can still integrate, but in significantly reduced numbers and with markedly reduced survival rates in vitro as well, possibly partly related to the mechanical and enzymatic disassociation required to prepare them [41].

Until recently, it was thought that donor photoreceptors were able to physically migrate and integrate into the recipient outer nuclear layer based on studies using immunofluorescent (IF) labelling of proteins that could only originate from the donor. However, recent studies have suggested that this is a relatively rare event in animals with some degree of photoreceptor preservation, and instead there appears to be some form of cytoplasmic exchange between the donor and host cells to account for the IF findings [12, 13, 39, 42–44]. However, in this review, for simplicity, we will continue to refer to ‘integration’ of photoreceptors even if this is not always the actual mechanism for disease rescue.

#### Retinal sheet transplant

Another way to transplant photoreceptors is as part of full thickness retinal sheet, ideally when they are about to start forming their synaptic connections, and when considerable plasticity exists (reviewed by Seiler and Aramant [34]). Indeed, foetal retinal tissue has been shown to be able to connect to mature neural tissue after transplantation into the brain [45]. Foetal retina also has lower immunogenicity containing less microglia (which migrate into human foetal retina from 16 weeks), fewer blood vessels and lower numbers of surface antigens than adult tissue [46–48]. In initial experiments in the early 1990s using microaggregates of retinal tissue, better preservation and growth of outer segments, albeit in rosettes, was demonstrated after transplantation into the subretinal space of RD1 mice as compared to single cells [49, 50]. Full thickness retinal sheets (including with attached RPE) have also been evaluated and shown to integrate with a degenerating retina and restore visual responses as shown in several rat models of retinal degeneration [51–53]. One of the problems of sheet transplantation is that sheet orientation is clearly important, but also budding photoreceptors axons have to migrate through the donor inner retina to reach the host bipolar cells. Partial thickness retinal sheets sectioned down to the photoreceptor layer using a vibratome or excimer laser have therefore also been evaluated [54–57], but these manoeuvres did not seem to improve connectivity and had lower transplant survival rates than full thickness grafts [57] probably relating to trauma.

Several patients have been treated with foetal retinal transplants delivered as microaggregates or sheets [58–60]. A Phase II trial was conducted in a group of ten patients (six with RP and four AMD) between 2002 and 2005 [61]. Vision improvement occurred in some patients and interestingly started about 6 months after surgery, corresponding to the time expected for the foetal cells to develop into functional photoreceptors and suggesting that the effectivity observed was not a short-term trophic effect.

In human trials, sheet transplants seem to have longer survival that microaggregates and probably single cells; a foetal retinal graft sheet in a clinical trial was observed to survive 3 years after the transplantation, while transplants in the form of microaggregates were no longer able to be detected [62]. However, systematic comparisons of photoreceptor sheets vs. suspension transplants in humans have not been published and it is not known whether one approach is better than the other regarding visual outcome or transplant survival.

The optimum age of foetal donor tissue for both sheet and single-cell photoreceptor transplants for these approaches is in the second trimester with obvious ethical and supply problems. Other sources of cells have therefore been sought and these are discussed later.

### Rescue of degenerating cells using cells of non-neural lineage

A variety of cell types have been shown to rescue photoreceptors in preclinical models of human retinal degenerative disease, including bone-marrow-derived hematopoietic and MSCs, adipocyte-derived cells, umbilical tissue cells and neural progenitor cells (NPCs). Although bone-marrow-derived MSCs can differentiate into cells expressing photoreceptor proteins when injected into the subretinal space, their ability to differentiate into functionally useful retinal cells is under debate. Their action is thought to be largely related to paracrine effects from neurotrophic factors (NTF) production. NTF are a family of proteins that participate in the regulation of the development, function, and survival of neurons and other cells in the nervous system [63, 64]. Early-phase clinical trials with encapsulated RPE cells producing ciliary neurotrophic factor (CNTF) suggested some photoreceptor protection in patients with retinal degeneration [65–67]. Although the effect of most factors on photoreceptor survival is indirect via microglia and Müller cells [68], red-green cones express the brain-derived neurotrophic factor (BDNF) receptor trkB and can directly respond to BDNF [69]. Transplants of rods in murine degeneration models slow cone degeneration [70]. This so-called rod-derived cone viability factor is a diffusible factor, synthesised by rods, and distinct from other known trophic factors [71].

As mentioned previously, RPE cells may also exert a significant effect on photoreceptor regeneration and survival by trophic effects alone, thought largely to be due to the production of pigment epithelium-derived factor (PEDF).

Delivery of cells to a subfoveal location requires foveal detachment, which could compromise the vision of the patient, particularly in the setting of retinal degenerative disease. Transplantation of NPCs subretinally has, however, demonstrated that they can migrate from the subretinal injection site quite extensively [72, 73], allowing the possibility that extrafoveal transplantation of some of these rescue cell types will have a more direct foveal effect not only from trophic factor release but also cell migration.

Many of these classes of cells also display variable immunomodulatory activities. The most well established is the suppression of immune responses and inflammation by bone-marrow-derived MSCs. Under various conditions, MSCs induce the expression of immune-modulatory proteins, including Ym1, insulin-like growth factor-1 (IGF-1), Th2-related cytokines, galectin-3, and class II major histocompatibility complex (MHC) antigens [74]. However, untreated MSCs in an in vitro rat retina-explant model transdifferentiated into microglial cells [75] and non-autologous MSC transplantation may induce an inflammatory reaction.

### The potential for in situ regeneration

Many species of amphibians and fish have a remarkable ability to regenerate damaged retinas. The source of cells for this appears to come from three main sources of resident stem cells dependent on species and age; the anterior retina in the ciliary margin zone, Muller cells (MC), and RPE [76–80]. Humans do not have these abilities; however, interestingly, the mammalian MC transcriptome overlaps significantly with that of RPCs and in vitro studies have identified a population of human MCs that are able to develop into retinal neurons, including those with photoreceptor markers [81–83]. Furthermore, on transplantation into a rodent model of photoreceptor degeneration, these MC-derived photoreceptor-like cells were able to migrate and integrate into the host outer nuclear layer, leading to an improvement in photoreceptor function as assessed by electroretinography [78]. The feasibility of such an ex vivo approach, however, is limited by their limited ability to self-renew and differentiate in vitro, their yet undetermined capacity to generate mature retinal neurons as well as donor tissue availability. If, however, the signalling pathways that regulate differentiation of these cells into desired phenotypes (e.g., photoreceptors, RPE) could be identified, a future hope would be that cell replacement could be initiated endogenously via gene reprogramming and differentiation of adult stem cells in situ. Indeed, recently, Jorstad et al. showed that by overexpression of the proneural transcription factor Ascl1 combined with the use of a histone deacetylase inhibitor, which altered the epigenetic profile of the MC genome, adult mice were able to generate retinal neurons from MCs in situ after retinal injury [84].

## Sources of cells for transplantation

A wide variety of exogenous cell sources for transplantation have been considered and are listed in Table 1. They can be broadly divided into adult tissue-derived stem cells, foetal-derived progenitor cells and pluripotent stem cell-derived cells. A summary of current and recent clinical trials is shown in Table 2.

**Table 1:** Cell sources for transplantation

Category	Type	Current or previous human trials in AMD?
Autologous ocular tissue	RPE patch +/− Bruch’s membrane and choroid	Yes [26, 27, 85, 86]
	Peripheral RPE as suspension	Yes [87]
	Iris pigment epithelium	Yes as cell suspension [88–90]
Foetal	Neuroretina	Yes as cells suspension and microaggregates [58, 59]
	RPE sheet	Yes [91, 92]
	Combined RPE and retinal sheet	Yes [61, 93]
	Retinal progenitor cells	Yes—See Table 2
Adult stem cells	RPE	To date only as an allograft of unsorted adult RPE [94, 95]
	Muller cells	Not as yet
	Ciliary margin zone stem cells	Not as yet
Mesenchymal stem cells		Yes—intravitreal and subretinal Table 2
Adipose-derived cells		Yes—Intravitreal (Table 2).
Umbilical tissue cells		Yes—cell suspension subretinally delivered via transchoroidal route (Table 2).
Embryonic stem cells	Differentiated to all retinal cell types	Yes as RPE cell suspension and sheet (Table 2).
Induced pluripotent stem cells	Differentiated to all retinal cell types	Yes as cell sheet Table 2

**Table 2:** Current and recent clinical trials focusing on cellular treatments for AMD and other outer retinal diseases

NCT number	Disease	Cells	Sponsor	Route	Planned completion	No. of patients	Phase	Results published?
*Human embryonic stem cell (hESC)-derived RPE suspensions*								
NCT01469832	SMD	hESC-derived RPE	Astellas Institute for Regenerative Medicine	Subretinal suspension	Completed	13		Yes for phase 1: safety shown [119, 212, 213]
NCT01344993	Dry AMD					13		
NCT01345006	SMD					13		
NCT03178149	Dry AMD				Uncertain	150	1/2	
NCT01674829	Dry AMD	hES-derived RPE	CHABiotech CO., Ltd	Subretinal suspension	April 2016	12	1/2	No
NCT02903576	Wet and dry AMD and SMD	ESC-derived RPE	University of San Paulo, Brazil	Subretinal suspension	June 2017	18	1/2	No
NCT01674829 and NCT1625559	Dry AMD and SMD	hESC-derived RPE cells	CHA Bio and Diostech, Republic of Korea	Subretinal injection	June 2015	12	1/2	No
NCT03046407	Dry AMD	hESC-derived RPE	Southwest Hospital, China	Subretinal injection	December 2017	15	1	No
NCT0274973	SMD and dry AMD							
NCT02286089	Dry AMD	hESC-derived RPE	Cell Cure Neurosciences Ltd.	Subretinal suspension	August 2017	15	1/2	No
*Pluripotent stem-cell-derived RPE sheets*								
NCT02590692	Dry AMD	hESC-derived RPE	Regenerative Patch Technologies, LLC	RPE monolayer on parylene sheet	September 2022	20	1/2	No
NCT01691261	Acute wet AMD	hESC-derived RPE cells	University College London	RPE monolayer on a 6 mm × 3 mm sheet on an polyester membrane	Uncertain	10	1	No
	Wet AMD	Autologous hiPSC-derived RPE	Kobe, Japan	RPE monolayer; 1.3 mm by 3 mm on own basement membrane	Temporarily suspended	6	1	Yes: 1st case presented
*Human CNS cells*								
NCT01632527	Dry AMD	Human central nervous system stem cells	StemCells, Inc.	Subretinal suspension	June 2015	15	1/2	No results unknown
*Encapsulated cells*								
NCT00447993	Late and early-stage RP	Human RPE cells contained in a capsule secreting CNTF	Neurotech Pharmaceuticals	Intravitreal insertion of capsule	Completed	65 and 68	2/3	Yes: neither study reached primary endpoint ^214^
*Bone marrow (BM), mesenchymal stem cell (MSC) and umbilical cell therapies*								
NCT01068561	RP	Autologous BM-derived MSC	University of San Paulo, Brazil	Intravitreal injection	Dec 2016	5	1	No
NCT01531348	RP	Autologous BM-derived MSC	Mahidol University, Thailand	Intravitreal injection	NK	10	1	No
NCT01736059	RP, dry AMD, diabetic maculopathy and retinal vein occlusions	Autologous CD34+BM stem cells	University of California, USA	Intravitreal injection	December 2017	15	1	Not for AMD
NCT01518127	Wet and dry AMD and SMD	Autologous BM stem cells	University of San Paulo, Brazil	Intravitreal injection	Jan 2013	10	1/2	Yes ^215^
NCT01560715						20		
NCT01920867	AMD/RP	Autologous bone marrow-derived stem cells	Retina Associates of South Florida, USA	Retrobulbar, Subtenon, Intravenous, Intravitreal injections	Aug 2017	300	1/2	No
NCT02709876	RP	Autologous BM-derived CD34+, CD133+, and CD271+Stem Cell	Stem Cells Arabia	Intravitreal injection	March 2019	50	1/2	No
NCT03011541	Various	Autologous bone-marrow-derived stem cells	MD Stem Cells	Retrobulbar, Subtenon, Intravenous, Intravitreal injections	June 2020	500	2	Not for AMD
NCT02659098	Dry AMD	Umbilical cord stem cells	Janssen Research & Development, LLC	Subretinal suspension via transchoroidal route	March 2022	255	2	Yes from phase 1
*Human foetal-derived cells*								
NCT02464436	RP	Human foetal-derived RPC	ReNeuron Limited	Subretinal suspension	September 2017	15	1/2	No
NCT02320812	RP	Human foetal-derived RPC	jCyte, Inc	Intravitreal injection	June 2017	28	1/2	No
NCT02868424	Dry AMD	Human foetal RPE cells	Nanjing Medical University, China	Subretinal suspension	Aug 2018	6	1	No

*SMD* Stargardt’s Macular Dystrophy, *RPC* retinal progenitor cells, *RPE* retinal pigment epithelium, *AMD* age-related macular degeneration, *RP* retinitis pigmentosa

For photoreceptor cells, as described above, the developmental stage and the environment the cells are grown in are vital to their ability to fully develop after transplantation and to engraft into the retina. Pluripotent stem cells (PSCs) are currently considered to be the optimum cell source for these but the potential of foetal-derived RPC will also be discussed.

PSCs could also provide a ready source of RPE cells. However, in contrast to photoreceptor transplants, transplanted RPE cells, virtually regardless of age or origin, readily extend apical villous process around host photoreceptors and phagocytose shed outer segment discs [15]. Autologous RPE cells from the peripheral retina are a viable source, but current methodologies of autologous transplantation require major surgery with significant risk and side effects and variable results. At least some of the variability relates to surgical trauma to the RPE from the donor site, as well as senescence and disease-carrying gene variants and as such alternative cell sources have been sought.

### Iris pigmented epithelial cells

Iris pigmented epithelial (IPE) cells are derived from the same embryonic cell line as RPE and are similar in many respects to RPE cells with apical/basal polarisation, microvilli, and the same type of tight junctions. Their appeal lies in the fact that they can be easily collected by surgical iridectomy and so could be autologous [85–90]. There are, however, several functional differences, although it is possible that IPE cells could acquire RPE properties when transplanted into the subretinal space in patients with AMD. Gene expression for intra- and extracellular retinal-binding proteins, which are essential for the visual cycle is lower in IPE cells than RPE cells [88]. In vitro IPE cells, although able to phagocytose photoreceptor outer segments, are less able to degrade them compared with RPE cells [89]. The level of expression of VEGF is also lower in IPE cells than in RPE cells [90], which may have consequences on choroidal restoration/health after transplantation but may also be beneficial in the context of wet AMD [85]. Suspensions of IPE have been evaluated in clinical trials with possible modest improvements in vision [91–93]. Ultimately, however, they suffer from the problems of senescence and the same disease-carrying genetic profile of the autologous donor.

### Foetal and adult RPE

Foetal RPE overcomes the problems of senescence but has obvious problems of ethical acceptability and supply. However, the recent finding that the human RPE contains a small subpopulation of cells that can self-renew and act as adult RPE stem cells [94] capable of producing large numbers of ‘new’ RPE cells is an exciting development [94, 95]. Transplantation of these created allogeneic RPE rescued vision in the RCS rat in a differentiation stage-dependent manner. Specifically, transplantation of an intermediate 4-week stage of RPE differentiation most consistently preserved vision compared to older or younger RPE cells [95].

### Foetal retinal progenitors

In vivo RPC exit the cell cycle and start differentiating into photoreceptors between 14 and 20 weeks of gestation and aside from ethical concerns, they would appear to offer a feasible source of cells for photoreceptor replacement. They also have relatively low immunogenicity and are not always rejected. Several studies have evaluated the use of disassociated foetal RPC for cellular transplantation [96] and at least two lines have been developed commercially and are in phase 1/2 studies in patients with photoreceptor dystrophies. ReNeuron have developed an interesting technology, whereby foetal RPC can be expanded at least 40-fold in hypoxic conditions akin to that experienced in vivo, overcoming many of the supply problems in obtaining these cells from week 16 to week 18 human foetuses. Publications have suggested that in vitro expanded RPC have some ability to differentiate and express photoreceptor markers [97–99], but this is limited and differentiation after transplantation appears to be dependent on host conditions particularly maturity, with transplant into immature retinas being more successful than mature ones [100−102]. Furthermore, cell migration into the outer retina is variable and the predominant markers expressed by transplanted cells are glial [103]. It is possible, however, that differentiation, migration and survival can be improved by alterations in delivery and the host environment [104, 105] and furthermore trophic and cytoplasmic transfer mechanisms still make these cells lines a potentially effective regenerative strategy.

### Pluripotent stem cells

Human PSCs can produce all the cell types needed for retinal regeneration (reviewed by Borooah et al. [106]). They are emerging as the preferred cell source because of their accessibility, expansion ability, and their ability to mimic retinal development with all its complexity to produce cells of the exact developmental stage required.

**Fig. 6:**
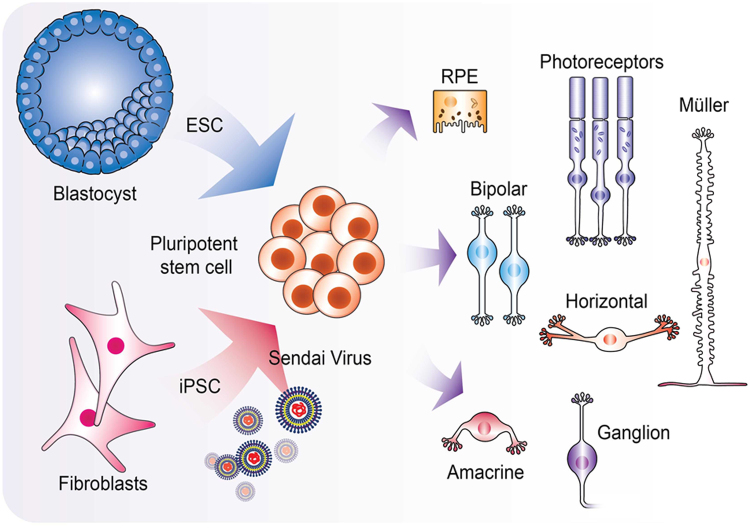
Schematic diagram showing the sources, and retinal differentiation potential of human pluripotent stem cells. Embryonic stem cells are derived from the inner cell mass of a pre-implantation embryo. Pluripotency can be induced in adult somatic stem cells by the delivery of key transcription factors that reprogramme the cells (delivered in illustration by non-integrating Sendai viruses)

The umbrella term PSC is most commonly used to describe two types of stem cells: embryonic stem cells (ESCs) and induced pluripotent stem cells (iPSCs). Both are defined by two key properties, namely an indefinite self-renewal ability and the capacity to give rise to any adult somatic cell type [107] (Fig. 6).

Human ESCs (hESCs) are derived from the inner cell mass of a preimplantation blastocyst. Several lines have been created since their discovery in 1998 and used to understand the mechanisms behind human embryonic development and congenital disease [108]. The use of hESC for research purposes is surrounded by a number of ethical issues and their use is prohibited in several countries. Their differentiated progeny express human leucocyte antigens (HLAs) that could result in graft rejection after transplantation, although this could be overcome by the creation of HLA-typed hESC banks, from which a best match could be selected for each transplant recipient.

In 2006, Yamanaka and Takahashi, building on the work of Gurdon, isolated four key transcription factors (gene expression ‘controllers’) that when expressed exogenously in somatic cells induced the formation of pluripotent cells in a process termed reprogramming [109, 110]. These ‘induced’ pluripotent stem cells (iPSC) share properties with ESCs, including the ability to self-renew and to be differentiated into the three germ layers [111]. Importantly, they can provide a source of autologous stem cells without the ethical concerns of ESCs. iPSCs not only provide a source of cells for transplantation but also present a unique opportunity to create in vitro disease models [112, 113], which can be used to understand disease pathology and drug discovery; particularly useful for retina where availability of patient-specific cells (i.e., photoreceptors and RPE) is only possible with invasive surgery or post mortem. Challenges still remain in finding the optimum strategy to ensure complete reprogramming (which can affect differentiation ability), maximising efficiency and minimising genetic changes during and after the process, but progress has been rapid and the technology is advancing at a pace.

Being autologous, however, also creates a potential problem for transplantation in that the cells will carry the same disease-causing gene mutations of the patient. This might not always be a problem in some diseases, particularly AMD, where onset is in later life, but even then gene editing may be useful to enhance integration or reduce recurrence. Current gene therapy approaches in the context of a patient with a known gene mutation uses viral vectors to transfect a corrected copy of the gene and a suitable promoter either in the cytoplasm or a ‘safe’ site in the genome in either the RPE or retinal cell type affected. These cells, being mature and non-dividing, only need this transfection event to occur once for prolonged protein expression. Conversely, in a PSC-derived cell line being delivered for transplantation, the optimum way of carrying out gene therapy would be to edit the genome at the PSC stage, to precisely correct for the mutation, thus correcting all derived differentiated cells thereafter. New gene editing technologies use specifically designed site-specific restriction endonucleases, such as Zinc Finger Nucleases (ZFNs), Transcription Activator-Like Effector Nucleases (TALENs), and clustered regularly interspaced short palindromic repeat/CAS9 RNA guided nucleases (CRISPR/Cas) [114−116] to excise the mutant part of the gene and simultaneously deliver a corrected copy, which then integrates into the cleavage point by homologous recombination.

The use of PSC also carries another concern namely tumour formation. Transplantation of undifferentiated ESC and iPSC, which could exist in a differentiating colony, can result in teratoma formation. In one study, transplantation of ESC-derived neural precursors into the subretinal space of a mouse model of RP resulted in teratoma formation in 50% of the mice within 8 weeks [117]. Sorting for markers that identify these remaining pluripotent cells, including TRA-1-60 and SSEA1 before transplantation is one way to reduce the risk of this. Yamanaka used four transcription factors OCT4, SOX2, KLF4 and c-MYC to reprogram cells and inserted these into the genome using an integrating retrovirus-derived vector. C-Myc is a potent oncogene and the process of transcription factor insertion and expression can cause genome mutations and dysfunction. Furthermore, human ESC-derived cell lines can exhibit phenotypic instability or develop altered gene expression, including variable X-inactivation with serial passaging in culture. Alternative strategies have been developed with the view of bypassing some of the issues outlined above. Protocols for reprogramming without c-Myc and using various combinations of other transcription factors have been tested [118]. Additionally, alternative methods exist that use non-integrative approaches, such as RNA-based Sendai virus, and non-viral delivery systems, including episomal vectors, messenger RNA and recombinant proteins with varying efficiency. Whatever the system chosen, ongoing vigilance is essential to detect chromosomal and genetic alterations in the cells used. Indeed, recently, an iPSC-RPE transplant trial was halted partly related to the finding of genetic changes in the iPSC-derived cell line. Three genes had been deleted, and there were mutations in three further genes, including one oncogene. However, reassuringly in the one patient treated in this trial and the patients treated in a large completed ESC-RPE transplantation trial, there have been no tumours reported [119, 120].

A third type of autologous PSCs can be produced by cloning. In this technique, termed somatic cell nuclear transfer, the nucleus of a donor adult somatic cell is transplanted into an enucleated oocyte to produce a near genetically identical cell to the original adult nuclear donor, akin to an ESC and as used subsequently to produce ‘Dolly the sheep’ [121]. These ESCs produced by nuclear transfer are more difficult to generate than iPSCs; however, the ability of the technique to replace aged or diseased mitochondria and the fact that they appear to be closer in terms of epigenetic and transcriptomic profiles to ESC may make this a useful approach for some applications in the future [122].

### Differentiation towards RPE and photoreceptors

On the basis of early publications describing differentiation of hESCs, all retinal cell types have now been differentiated from human iPSCs (hiPSC). Differentiation models development in vivo in which the initial cell mass, representing the epiblast divides into the three embryonic germ layers before the ectoderm differentiates further into neuroectoderm then retinal cell types [123−126]. At a cellular level, PSCs undergo stepwise differentiation through neural and retinal progenitor stages before differentiating to either RPE or neuroretinal cell types, all of which can be detected by specific immunofluorescent markers. Cells following an RPE fate show increasing MITF expression and neuro-retinal cells CHX10 expression, followed by cone-rod homeobox protein (Crx) and Recoverin expression as they develop into photoreceptors. In human PSCs, this process takes several months depending on culture conditions with cone markers appearing before rods [127, 128].

In vitro retinal differentiation protocols for PSCs can be broadly divided into default differentiation, in which cells differentiate to retinal lineages in the absence of extrinsic growth factors [129] and directed differentiation protocols, in which extrinsic transcription factors, proteins, and small molecules are added to direct the differentiation pathway along a specific course in a more efficient process both in terms of cell specificity and time course. Directed differentiation protocols are based on knowledge of basic principles of developmental neurobiology. It is known that retinal differentiation is controlled by a diverse set of signalling pathways that affect the identity of the resulting cell population. As a result, various exogenous factors have been used to direct the differentiation towards the desired retinal cell type. A number of studies have highlighted the importance of TGFβ, BMP, Wnt and Nodal signalling pathways in retinal fate commitment. Various research groups have used a combination of small molecules and recombinant proteins targeting these pathways in their differentiation protocols, including SB431542, Noggin, DKK1, LEFTY-A and Activin A [125, 130–132]. IGF1 has also been shown to augment differentiation towards RPE and neural retina [123, 128]. Some groups have investigated the effect of culture conditions on RPE differentiation, including using different growth substrates [133, 134]. The level of oxygen during differentiation has also been investigated as a possible modulator in cell differentiation. There is some evidence that hypoxic conditions during stem cell culture lead to favourable outcome in neuroectodermal and retinal differentiation [135−137].

iPSC lines vary greatly in their ability to differentiate into RPE and photoreceptors. These differences can be attributed to multiple factors. Variation in endogenous gene expression controlling differentiation have been shown to be one of the factors [138, 139], in which case a more tailored approached to differentiation protocols design may be required. Mutations in mitochondrial DNA are believed to introduce additional variability [140]. Other factors that have been shown to contribute to differentiation efficiency include DNA methylation, epigenetic memory, genetic background of hPSCs, and X chromosome inactivation, potentially leading to increased expression of oncogenes (reviewed by Ortmann et al. [141]). Recently, significant effort has been made towards driving PSCs towards a so-called ‘naive’ ground state, which is believed to be the only true totipotent type of cell with no differentiation bias. However, so far many studies have only partially managed to recapitulate ground state requiring additional study [142].

### Three-dimensional retinal culture

**Fig. 7:**
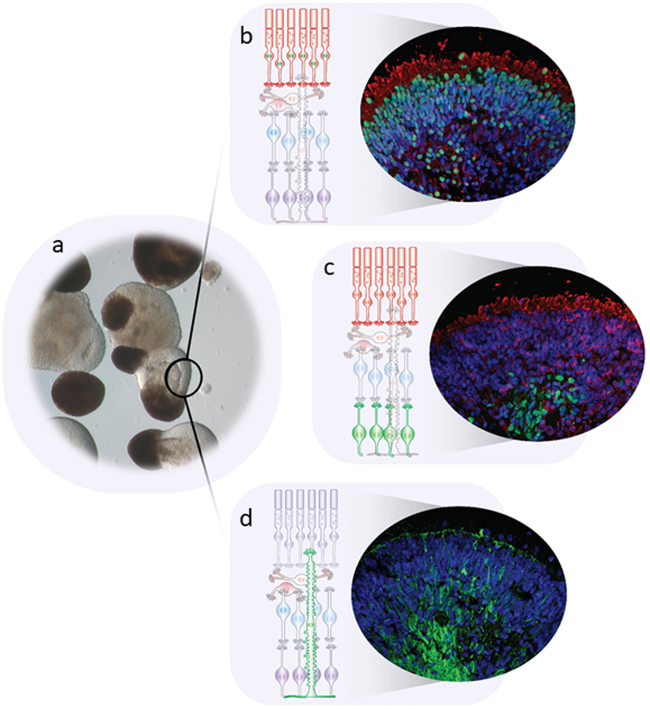
Retinal organoids with adjacent RPE 3D differentiation from human pluripotent stem cells. Optic vesicles with lamination (**a**). **b**–**d** show a diagrammatic representation of the laminated area with schematic antibody labelling, adjacent to actual antibody-stained sections. **b** Photoreceptors labelled with CRX (green) and Recoverin (red), **c** photoreceptors, Recoverin (red), and retinal ganglion cells, HuC/D (green), **d** Muller glia (green)

A major leap forward in photoreceptor differentiation was made in 2011 when Eiraku et al. showed that by suspending developing PSC in 3D culture conditions, the retina could be observed to arise from a neuroectodermal structure mimicking the anterior part of the primitive forebrain, with optic grooves protruding to form optic vesicles, and then invaginating spontaneously (rarely) to form a double walled optic cup structure, without the requirement for surface ectoderm [143]. Several laboratories have replicated this work now with hiPSC [128, 144–146] and the retinal ‘organoids’ produced shown to replicate the organised retinal lamination seen in vivo with all key retinal cell types identified and mature features observed, including photoreceptor outer segments not seen in 2D cultures [146, 147]. The technology has great potential for optimising the maturation of photoreceptor cells prior to transplantation, offers the possibility of retinal sheet transplantation as well as huge opportunities for improved disease modelling, toxicology and drug testing (Fig. 7).

### RPE characteristics

RPE cells can be easily recognised by their characteristic pigmented polygonal appearance from an early stage in culture and subsequently dissected out to purify and mature. Their maturity can be assessed by a variety of morphological, molecular and functional features. RPE-specific markers include those for phagocytosis such as MERTK and the basal marker BEST1. Apical membrane-associated markers include Na+/K+ATPase. Visual cycle markers include RPE65, LRAT and CRALBP [148]. Phagocytosis, trans-epithelial resistance and apical (PEDF)/basal (VEGF) polarity of growth factor secretion can all be tested as measures of function. The optimum age for transplantation is uncertain but generally cells are used once mature RPE markers are expressed.

### Photoreceptor recognition

Being non-pigmented, photoreceptor cells suitable for transplantation are less easy to identify. The intracellular markers used in vitro to identify photoreceptors such as CRX and NRL cannot be used to sort cells for transplantation using fluorescent- or magnetic-activated cell sorting [149]. Studies using animal models have sorted photoreceptors using transgenic fluorescent protein expression driven by promoters of photoreceptor genes; however, these are unlikely to be acceptable for human studies. The cell surface marker CD73 has been identified as marker for rod photoreceptors isolated from foetal mice [150−153] and has been shown to increase cell integration either alone [153] or in combination with CD24 [152]. Similarly, a panel of surface markers (CD73+/CD133+/CD24+/CD47+/CD15−) [151] has been shown to increase integration rates in mice compared to single-cell surface markers. The situation is as yet less clear in human PSC-derived cells, although Welby et al. have recently identified a cone biomarker panel (SSEA1−, CD26+, CD133+, CD147+) that positively enriches for human foetal L/M-opsin cones as well as a stem cell-derived cone photoreceptor population [154].

## Current PSC-derived retinal cells delivery strategies in investigation

Successful cell replacement does not equate solely to the ability to produce, and isolate the desired cell types, the cells also have to be delivered successfully to the subretinal space, survive and be able to replace the function of the degenerated cells. We now discuss the various approaches being evaluated to achieve this and the challenges faced.

### RPE replacement

RPE replacement, in particular, using PSCs has progressed relatively rapidly and two approaches are currently being considered for their transplantation: RPE cell suspension injection into the subretinal space and RPE sheet transplantation.

#### RPE cell suspension

**Fig. 8:**
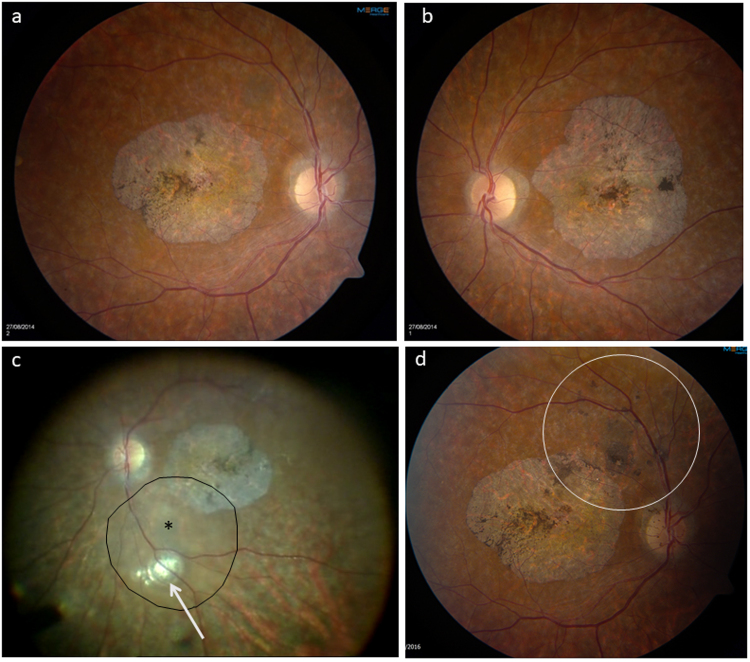
A 45-year-old male patient with Stargardt’s macular dystrophy with symmetrical atrophic maculae (**a**, **b**). Patient underwent vitrectomy with subretinal injection of a suspension of embryonic stem-cell-derived retinal pigment epithelial cells with an injection point superonasal to the foveal centre (**c**). (Injection point shown by black asterisk, area of subretinal bleb produced outlined by solid black line, with small subretinal air bubble indicated by white arrow: note image is intraoperative view with superior retina shown inferiorly). Nine-month postoperative appearance shows areas of subretinal pigment in the area of the original injection (white circle) (**d**)

Suspension of RPE cells transplanted into the subretinal space has rescued photoreceptors in numerous preclinical models of retinal degeneration [155]. Ocata Therapeutics, now Astellas, have carried out a number of phase I/II trials to evaluate the use of subretinally delivered hESC-derived RPE cell suspensions in patients with advanced dry AMD, Stargardt’s macular dystrophy and myopic macular degeneration. Systemic immunosuppression with tacrolimus and mycophenolate was used. Reassuringly safety and tolerability were demonstrated, and some patients showed improved vision in the injected eyes compared to the fellow untreated eyes; however, the usefulness of RPE transplantation in patients who already have poor vision owing to secondary photoreceptor atrophy is unclear. Fundoscopy of the patients showed gradually enlarging pigmented clusters subretinally in the area of injection, which could represent proliferating RPE cells but positive autofluorescence was not shown questioning their viability and function (Fig. 8). The clusters showed a predilection for focal areas of RPE atrophy in some patients, suggesting preferential growth in these areas but centrally in the area of gross atrophy there was no pigmentation produced. One possible explanation for this is that in AMD there are several abnormalities in BrM, including thickening, protein crosslinking, non-collagenous protein deposition, lipid deposition and advanced glycation end-product formation. A reduction in heparin sulphated proteoglycans [156] and an increase in tenascin-C and the chondroitin sulphate proteoglycan aggrecan, as well as a reduction in the levels of activated MMP-2 and -9 have been described in AMD, which may be responsible for some of the thickening seen related to impaired matrix degradation [157, 158]. All these changes result in a reduction in the normal integrins, the cell surface ligands that bind ECMs, including laminin, fibronectin, vitronectin, and which in turn affect the ability of transplanted RPE cells to adhere and form a monolayer, critical to their function and survival.

Various possibilities have been suggested to counteract this, including re-surfacing BrM to enhance RPE attachment. Research by Priore and colleagues showed that coating aged BrM with a mixture of laminin, fibronectin and vitronectin significantly improved cell adhesion, survival and proliferation and phagocytosis [159−161]. Another approach is to increase the surface levels of integrins. Long-term culture increases the expression levels of a-integrins, which has a positive effect on RPE cell adhesion to BrM [162]. Genetic manipulation can also be used to overexpress specific integrins specific to the altered BrM, and integrin function can also be modulated by modifying their activation state, including with manganese [158]. Ex vivo experiments using BrM from human cadaver eyes with AMD have documented impaired transplanted RPE survival in areas of RPE atrophy which could be improved using bovine corneal endothelial cell-conditioned media [163]. This was thought to work by altering cell behaviour and survival and not by altering BrM [163, 164]. Other ECMs and/or hydrogels could potentially be used in the same way and simultaneously improve cell distribution.

#### RPE sheet transplantation

Formation of a monolayer with apical-basal polarity and attachment to a surface are critical to RPE cell function and survival. Another approach to transplantation is, therefore, to deliver RPE cells as an intact monolayer on a carrier to replace BrM (reviewed by Hynes et al. and Delplace et al. [165, 166]). Use of a sheet scaffold may also prevent the BrM abnormalities from unfavourably altering the behaviour of the transplanted cells by acting as a barrier between them and the pathological BrM surface. Many different materials have been studied as potential BrM replacements. Both biodegradable/non-biodegradable and synthetic/natural membranes have been suggested. Carr et al. [167] have described the use of a vitronectin-coated polyester membrane to deliver an RPE monolayer to the subretinal space, while Lu et al. [168] used nanotechnology-manufactured parylene C scaffolds for RPE transplantation. A variety of other complex biomimetic non-biodegradable membranes that closely mimic native BrM and appear to optimise RPE cell growth and function have also been developed, although for delivery they may require a more rigid carrier [166]. Using electrospinning, various nanofibrillar materials have been investigated, including silk [169], polycaprolactone [169], polyimide [170], a combination of silk and polyethylene glycol [171], and an RGD-functionalized polymethyl methacrylate-co-polyethylene glycol methacrylate [172−174].

Two RPE sheet transplantation trials in humans are in progress both in patients with wet AMD of recent onset and both inserting RPE sheets grown from PSCs subretinally. The London Project to Cure Blindness (https://clinicaltrials.gov/ Identifier: NCT01691261) uses hESC-derived RPE on a polyester sheet. The Riken Institute in Japan (JPRNUMIN000011929) used hiPSC-derived RPE transplanted on its own type 4 collagen layer without the use of any artificial scaffolds. The Riken trial has been halted partly due to concerns regarding the genomic integrity in the donor cells but has reported results in one patient to date. Safety and tolerability have been shown and long-term cell survival suggested by graft appearance at 6 months [120]. The protocol is currently being revised to use HLA-matched allogeneic iPSC-derived RPE cells [175]. Further results are eagerly awaited.

It is useful also to reflect on some of the findings from previous work using autologous RPE cell patches. Postoperative proliferative vitreoretinopathy (fibrosis), induced partly by the retinotomy site to introduce the patch was a problem, but it is hoped that the avoidance of a donor retrieval site in the eye will reduce this. Sheet transplantation requires a significantly larger incision than suspension injection but multiple sheets rather than single large ones could be transplanted to cover larger areas. Autologous work has also suggested that peripheral retinotomies may be better tolerated than posterior ones as used in both of the current sheet trials and this is something that could be explored albeit with more extensive surgery required [176]. Another factor unaddressed at present is the role of a choroidal circulation in RPE sheet transplants. Generally in grafts for wet AMD, the area of choroidal neovascularisation is removed, which is accompanied by the adjacent choroid. Autologous vascularised graft survival is highly dependent on the re-establishment of a choroidal circulation from the periphery of the graft [177]. It is unknown how important this will be in non-vascularised RPE grafts. OCT angiography may be particularly revealing in assessing this aspect as has been used successfully with autologous RPE/choroidal grafts [177].

### Photoreceptor replacement and regeneration

To date, there have been no clinical studies with PSC-derived photoreceptor cells, although this will likely occur soon based on a number of compelling animal studies. Human trials have commenced with foetal-derived RPC and previously carried out with foetal-derived retinal sheets.

#### Ongoing developments in transplantation of photoreceptors

Studies in animal models of retinal degeneration have now demonstrated that transplanted photoreceptor cell suspensions can restore visual function, including using PSC-derived cells albeit with reduced effect. Experiments can be divided into those with remaining photoreceptors and a completely absent photoreceptor layer.

Studies in animals with some surviving photoreceptors have had several key findings. ‘Integration’ (bearing in mind that some of this is secondary to cytoplasmic transfer of material with cell rescue rather than actual integration as mentioned previously) is very dependent on the subretinal microenvironment and this can affect the survival, morphology and functionality of transplanted cells (reviewed by Pearson et al. [19]). Both the causative genetic mutation and stage of the degenerative process are important, meaning that in clinical translation of this technology in the future the stage of the disease will be critical. It can also influence the propensity for cones or rods to successfully ‘integrate’; cones are more likely to ‘engraft’ in cone-depleted retinas; however generally cones have been shown to integrate in significantly lower numbers than rods [42], which poses a further challenge for possible macular photoreceptor replacement in AMD. Conversely, most mutations causing RP affect rods primarily and cones die secondarily in the later stages. Rod transplantation could therefore rescue cones from dying and has been demonstrated in animal models of RP [42, 178, 179].

The number of successfully ‘integrating’ cells can be increased by a variety of treatments to the host environment, including temporary disruption of the host external limiting membrane, digestion of disease-associated ECM, especially chondroitin sulphate proteoglycans using chondroitinase ABC and also potentially by modulation of glial cell hypertrophy/activation and matrix metalloprotease activity [180−182]. Despite these successes, survival rate of transplanted cells has been generally low, <5% and reduces with time. Injection of cells in hydrogels and with the addition of selected ECM molecules may enhance this. Survival of donor photoreceptors can also be improved using anti-apoptotic factors such as X-linked inhibitor of apoptosis protein (XIAP) and immunosuppression [183, 184] (see later).

Transplantation in animals with a completely degenerate outer retina and no remaining photoreceptors (as may be the case at the fovea in advanced AMD) results in subretinal cell clumps in close contact with the remaining inner nuclear layer with limited migration into the host. Synapse formation, correct orientation with respect to the RPE and visual responses have however been demonstrated and related to the number of surviving cells [44, 185, 186]. Photoreceptor survival and connection may be enhanced when cells are presented subretinally in a range of biodegradable synthetic polymers and this is another strategy being investigated. In actual fact only a relatively small number of functioning photoreceptors may be needed to restore vision. Gene therapy experiments in the Gnat1−/− mouse model of congenital stationary night blindness have indicated that navigative vision could be detected with only 25,000 functioning rods although approximately 150,000 functioning rods are necessary to generate a reliable scotopic ERG response [14]. Exactly how this relates to humans, especially foveal vision however is very unclear. There are further barriers that may interfere with successful visual restoration despite successful cell survival. These include synaptic connection to the anatomically intact inner retina, function of the transplanted photoreceptors, and the synaptic reorganisation of the inner retina that occurs with very advanced outer retinal atrophy. Indeed in animal models of advanced outer retinal degeneration caused by photoreceptor degenerations, synaptic connections between bipolar cells and amacrine cells are lost [187]. Plasticity after restoration of an afferent input however may occur and work with electronic retinal implants in patients with advanced AMD may be revealing in this context [8].

**Fig. 9:**
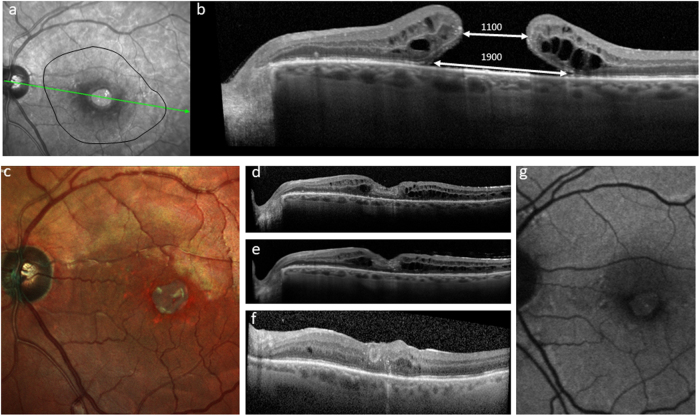
66-year-old male patient who developed a large macular hole with a previous macular involving retinal detachment. Despite a large diameter internal limiting membrane peel at the initial retinal reattachment surgery, the patient had a persisting macular hole after otherwise successful surgery (**a**, **b**: ILM peel area shown as black line). The patient underwent a free autologous transplantation of a patch of retina from just above the superotemporal arcade which was positioned within the macular hole rim. The day 1 postoperative appearance is shown in (**c**), and SDOCT at 2 weeks (**d**) and 6 weeks (**e**). Note the disorganised inner retina within the graft site but more normal appearing outer retina. SDOCT at 6 weeks following silicone oil removal showing an intact ellipsoid line (**f**.) Autofluorescent image taken at 6 weeks following oil removal (**g**) showing visible autofluorescence centrally. The patient displayed fixation over the graft with a visual acuity of logMAR 0.8

One other interesting development, which although not directly related to macular degeneration may have implications for retinal regeneration, has been a case report of a free autologous retinal patch transplanted from the peripheral retina to the fovea in a patient with a prior retinal detachment and large macular hole. The patient regained a visual acuity of 0.6logMAR with gradually improving retinal sensitivity and the authors postulated that the free graft played some role in retinal regeneration [188]. Further studies are needed but several surgeons have repeated the technique with visual benefit (Fig. 9) and further reports are imminent (personal communication).

#### Foetal-derived retinal progenitor cells

The exact mechanism of action of human RPC transplantation is unclear at present as discussed previously. They have rescued photoreceptor death in a number of animal models and current data suggests it may be chiefly neuroprotective with growth factor release. The transplanted cells may also differentiate into, attract or retain neuroprotective glial cells.

At least two human trials are in progress in the USA using foetal-derived RPC. ReNeuron (Bridgend, Wales, UK) have a Phase I/II open-label, dose escalation clinical trial in progress on 20 patients with RP to evaluate the safety, tolerability and preliminary efficacy of a subretinal injection of RPC. jCyte, Inc (Newport Beach, CA, USA) [189] have a phase 1/2 study of an intravitreal injection of RPC in patients with RP. Results from both trials are awaited and if successful these may be extended to patients with AMD.

#### Retinal sheets

As mentioned previously foetal retinal sheet transplantation has been carried out in adults with advanced AMD and retinal sheet transplantation may be a promising strategy especially in focal disease such as AMD. PSC-derived retinal organoids are an exciting development in this regard as they offer the possibility of deriving retinal sheets for transplantation of any developmental stage and in practical quantities. Takahashi and colleagues have used the ~2 mm optic vesicles derived from hESCs, sectioned them into halves and transplanted them into murine and primate models of outer retinal degeneration [190, 191]. They were able to demonstrate synaptic connection of the transplanted photoreceptors with the host in the halves that were transplanted by chance in the correct orientation, with functional recovery of vision in the mouse model tested. Future challenges relate to optimising the delivery and age of transplant, and increasing the size or number of grafts to improve the overall chance of direct integration. It would be also advantageous to enhance differentiation conditions toward a photoreceptor lineage, or even more specifically a cone lineage rather than inner retinal cells. Some progress has already been made in this direction by Zhou and colleagues, who have developed an in vitro method of differentiation of hESCs in S− and M-cones with evidence of connecting cilium and outer segment formation after 60 days [192].

### Combined RPE and photoreceptor transplantation

In advanced AMD, both RPE and photoreceptors would need to be transplanted. RPE often grows adjacent to neural retina in organoid models, but not as a continuous layer underlying the developing photoreceptors as occurs in vivo. It may be possible to develop culture conditions that encourage RPE growth and it has recently been shown that 0.5% alginate increases RPE growth alongside neural retina [193]. Alternatively, it may be possible to use tissue engineering to produce a combined transplant of an RPE monolayer on a biomimetic membrane with an additional biodegradable scaffold for a photoreceptor layer or retinal organoid-derived sheet. Similarly, structures including cells could be printed as a 3D construct [194]. There are as yet no reports of studies with these approaches.

### Choroidal replacement

There has been relatively little investigation into choroidal replacement in AMD regenerative strategies. Replacement of healthy RPE may rescue a failing choroid by VEGF and other growth factor release; however, there is evidence that choroidal endothelial cells (ECs) are lost early in the pathogenesis of AMD and as mentioned previously with autologous RPE transplants, choroidal reperfusion is essential for graft survival. Developing a procedure to replace these cells would be an important consideration in advanced cases of AMD—both wet and dry. Human iPSC-derived ECs have been made and studies with human umbilical vein-derived ECs suggest that if grown with RPE cells, ECs may acquire choroidal characteristics [195]. Choroidal ECs have also now been successfully generated from iPSCs [196, 197]. Decellularised human choroid with preservation of the architecture of the acellular vascular tubes has been successfully prepared and successfully seeded with immortalised human and primate ECs. Potentially a decellularised choroid and BrM may provide a good surface for iPSC-derived RPE and EC growth to produce a trilayer graft.

### Considerations for immune rejection

The subretinal space is considered to be a relatively immune-privileged site [161]; however, this depends on the integrity of the outer retinal blood barrier with an intact RPE layer [164]. There appears to be a lower propensity to rejection in subjects with dry AMD compared to wet AMD [198]. In mice, long-term (but not short term in some disease models at least [147]) allogeneic photoreceptor transplant survival requires immune suppression [164]; and in humans the need for long-term immune suppression would be a major problem in the elderly AMD population receiving allogeneic transplants. Autologous iPSCs present a way around this problem but the challenge of creating patient-specific cells for every person would be a very major undertaking. Although MHC expression is low in many types of stem cells, differentiated tissue expresses MHC, and this expression causes immune rejection. Recently, it has been shown in primate eyes that matching donors with recipients for HLA-A, HLA-B, and HLA-DRB1 prevents an immune response and would mean that banks of HLA-matched iPSC-derived cells could be usefully created [199]. However, even autologous mouse iPSCs can induce an immune response, akin to an autoimmune reaction [200] and only time will tell what will occur in human transplantation. Certainly, minimising surgical trauma will be important to reducing the activation of innate immunity (e.g., natural killer and dendritic cells), which is important on its own in rejection and which can in turn activate adaptive immune responses. Furthermore, minimising the use of implanted scaffold materials that can induce inflammation will be important. Other possible approaches would be immune tolerance induction by, for example, reducing MHC class II expression using small-interfering RNAs and other genetic manipulations of MHC expression.

### Host cell rescue

Bearing in mind that many cell types can have cell rescue effects, and conversely many have the potential for further differentiation into retinal cell types after transplantation, there are several cell lines being investigated, which are specifically being used for host cell rescue and undergoing clinical trials in patients with a variety of retinal diseases, including AMD (Table 2). Cells have been injected intravitreally, both in capsulated form and in a suspension.

Neurotech (Cumberland, RI, USA) have used a cell capsule implanted into the vitreous cavity and anchored at the pars plana containing human RPE cells genetically modified to overexpress CNTF and encapsulated in a polyethylene terephthalate scaffold. Phase I and II trials in patients with AMD (and RP) have shown the device is well tolerated but evidence of efficacy was less clear and the technology is now being considered for other applications, including to deliver anti-angiogenic agents in AMD (NCT02228304).

A wide variety of trials using MSC and other related cells are in progress. Early phase trials at the University of Sao Paulo, using autologous bone marrow-derived stem cells intravitreally in patients with advanced RP, showed some efficacy on macular oedema, but after 1 year, the effects were no longer evident [201]. The University of California has carried out a phase1 study using intravitreally administered autologous CD34+bone marrow stem cells in a range of retinal diseases, including advanced dry AMD. Preliminary clinical findings from the ongoing Phase I trial patients have showed some possible benefit [201−203].

Subretinal injection of human umbilical tissue-derived cells (hUTCs) (Palucorcel, CNTO-2476) have been evaluated in a phase 1/2 trial in 35 patients with advanced dry AMD. These cells are derived from extraembryonic mesoderm and can be expanded significantly in vitro but are not classed as stem cells and do not differentiate into other cell types. hUTCs have been shown to secrete several key neurotrophic factors that rescue RPE cell and photoreceptor function, including phagocytic dysfunction and have also been shown to promote synaptogenesis via thrombospondin family proteins [204, 205]. Visual acuity improved in several of the treated eyes compared to their fellow eyes but no changes in geographic atrophy extent or progression were observed. Retinal detachment occurred in 17% of the patients although whether these detachments were purely rhegmatogenous or partly tractional is unclear. Interestingly, the cells were injected using an ab externo approach to the subretinal space, rather than transvitreal as in most other trials, using a microcatheter passed transclerally and advanced under direct view to the paramacular area. Transchoroidal delivery avoids the problem of reflux of cells into the vitreous cavity and potentially retinal trauma, although in the trial retinal perforation occurred in 37% of patients and the method of delivery is being redesigned for the next phase of trials.

Preclinical studies using the RCS rat showed that subretinal transplantation of human NPCs derived from human prenatal cortex resulted in long-term rescue of visual function [72, 206, 207]. It was thought that their action related to their ability to phagocytose photoreceptor outer segments as well as other trophic effects [208]. Stemcells.inc completed a phase I clinical trial in patients with dry AMD and a phase 2 trial commenced but was terminated in 2016 (https://clinicaltrials.gov/, #NCT02467634). Trial results have not been reported and there appears to be no further studies planned.

Adipose tissue-derived cells have been used in several trials and are also being used in non-trial situations by private clinics. Unfortunately, recently three patients were reported who developed severe visual loss with a variety of problems, including ocular hypertension, haemorrhagic retinopathy, vitreous haemorrhage, combined traction and rhegmatogenous retinal detachment, and lens dislocation after intravitreally delivered cells [209].

## Conclusion

The dream of replacing or rescuing old, dead or dying cells with new young ones is appealing and the developments in the field are very exciting. However, the path to an effective safe cellular therapy that can be used routinely in patients for late-stage AMD is likely to be a long one. IPSC technology and 3D retinal organoid production have been huge recent steps in this path, but several aspects of cellular therapies outlined in this review need further refinement. The optimum cell sources and age of cells used for transplant need to be identified, cells have to be produced in the numbers required in GMP and Xeno-free conditions, and they then need to be sorted so that the desired cell types are isolated before transplantation. Cell delivery needs further thought as well. Cell sheet transplants require large retinotomies with their associated risks of PVR and subretinal fibrosis, which could not only cause surgical complications but also affect integration of the graft. Fibrosis has been a major challenge in several transplant disciplines and lessons could be learned across disciplines. Cell suspensions have problems of cell death during delivery, reflux during injection and a less than perfect cellular distribution after injection aside from RPE adhesion problems, all potentially aided by tissue engineering approaches. Cellular integration and survival after delivery also need improving perhaps aided by pro-survival agents added to the transplanted cells including potentially the use of combined gene therapy.

Preclinical testing would be easier to interpret if there were representative, near human size and accepted animal models of AMD. The commonly used model of the RCS rat is rescued by a wide variety of disparate cell sources and does not represent AMD very closely. Rabbit and pig models, although having visual streaks, lack foveas and in the case of rabbits are merangiotic, while primate models, as well as having ethical concerns and expense, do not have the problems of cell adherence to BrM seen in aged human subjects.

Unmasked fellow eye control phase I studies have established the safety of many treatments, but evidence of unequivocal, reproducible and sustained efficacy needs to be demonstrated. Indeed, there are several limitations to using these studies to suggest efficacy in terms of asymmetrical disease and progression, protective cytokine release with surgery (albeit usually short term) and learning effects in terms of eccentric fixation in worse fellow eyes [210, 211] and randomised masked studies are required to prove effect. The trials that have been conducted to date however have been essential first steps. Theory and laboratory work can only advance in the field so far and it is hoped these early trials are the first steps to proven effective and safe therapies.
